# Hyperglycemia and Endothelial Dysfunction in Atherosclerosis: Lessons from Type 1 Diabetes

**DOI:** 10.1155/2012/569654

**Published:** 2012-02-14

**Authors:** Steven Daniel Funk, Arif Yurdagul, A. Wayne Orr

**Affiliations:** Departments of Cell Biology and Anatomy and Pathology, LSU Health Sciences Center, Shreveport, LA 71130, USA

## Abstract

A clear relationship between diabetes and cardiovascular disease has been established for decades. Despite this, the mechanisms by which diabetes contributes to plaque formation remain in question. Some of this confusion derives from studies in type 2 diabetics where multiple components of metabolic syndrome show proatherosclerotic effects independent of underlying diabetes. However, the hyperglycemia that defines the diabetic condition independently affects atherogenesis in cell culture systems, animal models, and human patients. Endothelial cell biology plays a central role in atherosclerotic plaque formation regulating vessel permeability, inflammation, and thrombosis. The current paper highlights the mechanisms by which hyperglycemia affects endothelial cell biology to promote plaque formation.

## 1. Cardiovascular Disease and Diabetes Mellitus

Treatment of cardiovascular disease (CVD), manifesting in the form of myocardial infarction, stroke, and peripheral artery disease, represents one of biomedical sciences best success stories over the past several decades [1, 2]. Through clinical trials, epidemiology, and basic science, we have identified a host of risk factors and designed drugs targeting these risk factors that improve patient survival. The cholesterol-lowering statin family of therapeutics reduces the 5-year risk of cardiovascular-associated mortality by ~25% in patients with a history of prior CVD [3]. However, statins have not shown similar protection in patients without a prior history of CVD [4, 5], and CVD remains the leading cause of death in developed countries [2, 6]. Furthermore, the current obesity epidemic threatens to worsen the incidence of CVD in the coming years, undoing the progress we have made to this point [7].

More than 80% of the CVD-associated death and disability is attributed to atherosclerosis, the excessive accumulation of lipids, cholesterol, inflammatory cells, and connective tissue in the vessel wall [8, 9]. While clinically silent for decades, atherosclerotic plaques can grow to occlude the vessel lumen reducing blood flow to target tissues [8, 9]. Although this form of vessel occlusion can result in significant discomfort (e.g., angina pectoris), clinical events most often result from thrombus formation due to plaque deterioration or rupture resulting in a rapid cessation in blood flow to target tissue. Theories concerning the pathogenesis of atherosclerosis have changed over the years, maturing concomitantly with our understanding of vascular biology. We now know that atherosclerosis is a chronic inflammatory disease with multiple risk factors, such as hypercholesterolemia, dyslipidemia, diabetes, hypertension, and smoking, all playing roles in propagating the local inflammatory response [9, 10].

**Figure 1:**
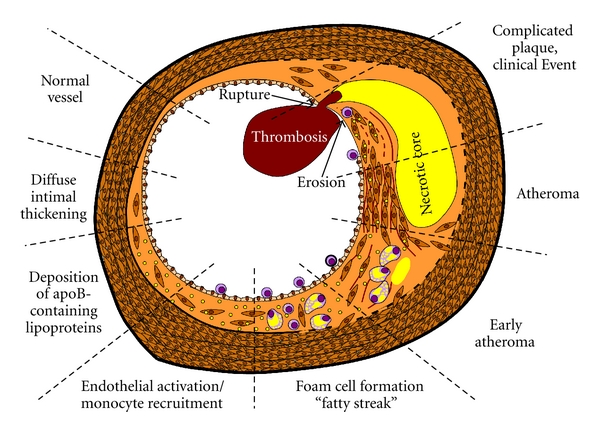
Stages of atherosclerotic plaque formation. Early apoB-containing lipoprotein accumulation and monocyte binding drive the early stages of plaque development forming fatty streaks in the vessel wall. While these processes continue in advanced atherosclerosis, monocyte cell death, smooth muscle recruitment, and matrix deposition are hallmarks of atheroma formation. Superficial plaque erosion and rupture of the smooth muscle-rich fibrotic cap cause plaque-associated thrombosis culminating in a clinical event.

Mouse models resulting in hypercholesterolemia, such as the ApoE knockout mouse and the low density lipoprotein (LDL) receptor knockout mouse, have allowed us to better understand the pathogenesis of atherosclerotic plaque formation and gain mechanistic insight into various biochemical pathways in mediating this response [11]. Atherosclerotic plaque production in both mice and humans localizes to discrete regions of the vascular tree that experience alterations in blood flow, such as vessel curvatures, branch points, and bifurcations [12]. Coupled with endothelial cell culture models, we now understand that shear stress, the frictional force generated by flowing blood, exerts a protective effect on the vascular endothelium to limit inflammation, thrombosis, and endothelial turnover [12, 13]. In contrast, turbulent flow promotes endothelial permeability and proinflammatory responses. Increased endothelial permeability and altered intimal matrix protein composition promote deposition of apoB-containing LDL particles within the intimal matrix (Figure 1) [14, 15]. Oxidation of LDL within the vessel wall enhances its proinflammatory properties resulting in local endothelial cell expression of inflammatory proteins (e.g., intercellular adhesion molecule (ICAM-1), vascular cell adhesion molecule (VCAM-1)) [8, 16]. Monocytes target to these regions of local inflammation and engulf the lipid deposits forming foam cells (Figure 1). These early plaque precursors, visible histologically as fatty streaks, accumulate smooth muscle cells, necrotic foam cell debris, lipids, and ECM proteins as they transition to advanced atherosclerotic plaques [8, 9]. While current methods to detect atherosclerosis rely on plaque stenosis, more than 60% of myocardial infarctions are caused by plaques showing less than 50% stenosis [17]. Consistent with this, plaque growth alone rarely results in vessel blockage as blood vessels adapt by expanding to maintain lumen size and affected tissues promote new blood vessel growth (termed angiogenesis) to restore blood supply [8]. Rather, cardiovascular events most often result from thrombus formation and acute vessel occlusion following plaque rupture or superficial plaque erosion (Figure 1) [18]. Plaque rupture is hindered by the plaque's fibrotic cap, a smooth muscle cell and extracellular matrix-rich region overlying the thrombotic necrotic core. Local smooth muscle cell apoptosis and enhanced matrix proteolysis by leukocyte-derived proteases weaken the cap enhancing the likelihood of plaque rupture; this is termed the “vulnerable plaque” [18, 19]. While plaque rupture accounts for approximately 70% of all thrombotic CVD events, the remaining 30% occurs following superficial plaque erosion resulting in the loss of the protective endothelial cell layer and exposure of the highly thrombogenic intimal matrix (Figure 1) [18, 19].

### 1.1. Diabetes Mellitus and CVD

The ancient Greek physician Aretaeus of Cappadocia used the term diabetes to refer to diseases associated with the excess production of urine [20]. The English physician Thomas Willis coined the term diabetes mellitus, literally translated “honey-sweet diabetes,” in 1645 in reference to the sweet-tasting urine of one of his patients [20]. Today, the term diabetes mellitus refers to the family of metabolic conditions associated with the loss of normal glucose regulation resulting in hyperglycemia. In 2000, there were 171 million diabetics worldwide comprising 2.8% of the population; this number is projected to reach 366 million (4.4% of the population) by 2030 [21]. Type 1 diabetes, also known as insulin-dependent diabetes mellitus (IDDM), typically results from autoimmune destruction of pancreatic islet cells responsible for insulin secretion within the first few decades of life. In contrast, type 2 diabetes, or non-insulin-dependent diabetes mellitus (NIDDM), involves progressive insulin resistance as target tissues become insensitive to insulin resulting in chronic hyperglycemia and hyperinsulinemia. Chronic hyperglycemia in patients is measured by determining the level of glycated hemoglobin (HbA1c). Since red blood cells have a 3-month life-span, HbA1c values provide an estimation of average blood glucose levels over time [22]. In healthy patients, HbA1c levels range from 4.0 to 5.9% corresponding to a blood glucose level of 68 to 123 mg/dL. An HbA1c level greater than 6.5% (140 mg/dL blood glucose) is used as a criterion for the diagnosis of diabetes. However, the HbA1c level does not provide any information concerning periodic spikes in hyperglycemia which may be just as harmful as a sustained higher average glucose. As such, continuous blood glucose monitoring systems are becoming more and more common.

Diabetics have a 2- to 4-fold higher risk for cardiovascular events [23], and nearly 80% of diabetes-associated deaths are caused by CVD [24]. As such, diabetes is regarded as a coronary heart disease risk equivalent, meaning that the risk for CVD is the same as an individual with a previous CVD event [25]. The enhanced CVD risk in diabetic patients is larger for women than men because women generally enjoy a protection from CVD during their reproductive years, and this protection is lost in diabetics [26]. Type 1 and type 2 diabetic patients show a similar atherosclerotic plaque profile with an increase in necrotic core size and a decrease in the fibrotic cap size [27, 28]. However, type 2 diabetics show enhanced atherosclerotic plaque burden with more distal plaques compared to type 1 diabetics [28]. Consistent with this disparity, type 2 diabetes represents a multifactorial proatherogenic effect involving various aspects of metabolic syndrome, a combination of hyperglycemia, hyperlipidemia, obesity, and hypertension [29]. Obesity, particularly abdominal obesity, results in enhanced expression of systemic circulating proinflammatory cytokines expression and reduced levels of protective factors such as adiponectin [30, 31]. Furthermore, obesity is linked with the hyperlipidemia and hypercholesterolemia classically associated with atherosclerotic plaque formation [30, 31]. Due to the presence of these confounding factors, the specific role of hyperglycemia on atherosclerotic plaque formation in type 2 diabetics has been difficult to discern.

Despite these complications, considerable data exist linking hyperglycemia alone to accelerated atherosclerosis in type 1 diabetics, where these confounding atherogenic factors are often absent. Children with type 1 diabetes show enhanced carotid intimal-medial thickness (IMT) compared to nondiabetics [32]. Postmortem studies of young patients and children with type 1 diabetes show enhanced fatty streak formation in the absence of dyslipidemia suggesting that hyperglycemia is an independent risk factor for early plaque development [33, 34]. Animal models of type 1 diabetes include pancreatic *β*-cells destruction by the DNA-alkylating mechanism of streptozotocin [35], targeted autoimmune destruction of *β*-cells due to transgenic expression of viral proteins (RIP-GP mice) [36], and decreased proinsulin production and processing due to mutations in the insulin 2 gene (Ins2Akita (*Mody*) mouse) [37]. Diabetes alone in these animals does not appear to be sufficient for significant atherosclerotic progression. However, when coupled with genetic modifications to the LDL-R or ApoE genes to induce a more human lipoprotein profile, diabetic mice show enhanced atherosclerotic plaque production compared to their nondiabetic littermates [38–40]. Hyperglycemia is strongly associated with early fatty streak formation in atherosclerosis-prone mice, while progression to advanced atherosclerotic plaques requires dyslipidemia [41]. However, diabetes in these models is often associated with elevated hypercholesterolemia compared to nondiabetic controls, making it difficult to elucidate whether the observed effects on atherogenesis are due to hyperglycemia specifically.

Clinical trials have both corroborated and complicated the role of hyperglycemia in atherosclerosis. The DECODE trial showed an association between hyperglycemia and impaired glucose tolerance and CVD [42, 43]. However, studies to elucidate whether tight glycemia control reduces CVD risk have produced mixed results, likely attributed to the timing of the treatment and the inclusion of low CVD risk or high CVD risk patients. The Diabetes Control and Complications Trial (DCCT) examined the effect of tight glycemic control in young type 1 diabetics with low CVD risk. Within the initial time period of the study, the authors demonstrated reduced microvascular complications but only demonstrated an insignificant (41%) reduction in CVD events [44]. However, the event rate for CVD in this population was very low. In the Epidemiology of Diabetes Interventions and Complications (EDIC) study, a 10-year followup of the DCCT trial, the investigators found a significant 42% reduction in CVD events [45]. The UK Prospective Diabetes Study (UKPDS) examined the role of tight glycemic control in newly diagnosed type 2 diabetics. Similarly, this group found a nonsignificant reduction in CVD events (16%) during the course of the trial [46] but a significant reduction in CVD events in a 10-year follow-up study [47]. However, clinical trials examining stringent glycemic control in patients with an already high CVD risk (Action to Control Cardiovascular Risk in Diabetes (ACCORD), Action in diabetes and Vascular disease: Preterax and Diamicron MR controlled evaluation (ADVANCE), Veterans Affairs Diabetes Trial (VADT)) showed no beneficial effect on CVD [48, 49]. Furthermore, the ACCORD trial demonstrated an increase in CVD-associated death in the intensively controlled group (HbA1c < 6); this effect has been attributed to enhanced weight gain, increased insulin injections, and increased incidence of hypoglycemia. These differential responses may result from the timing of glycemic control, as patients in the VADT trial that were within the first 12 years of diagnosis showed a benefit in the intensive treatment arm [50]. Taken together, data from clinical trials suggest that glucose control early following the diagnosis of diabetes confers protection that is often not evident for decades despite the cessation of differential treatment; consistent with the hypothesis that hyperglycemia plays a larger role in plaque initiation than plaque progression.

## 2. Endothelial Cell Activation and Dysfunction in Atherosclerosis

**Figure 2:**
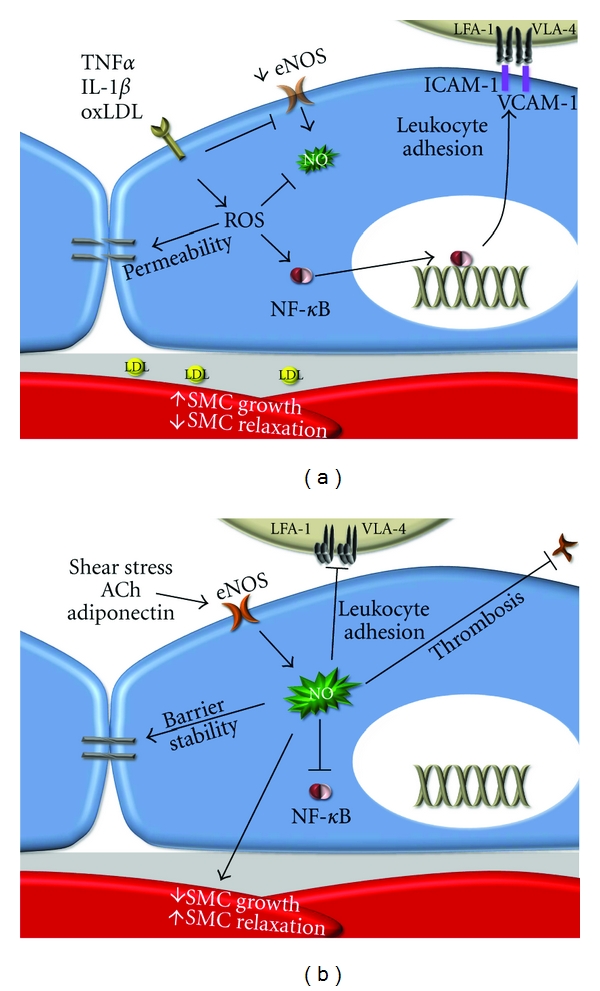
Interplay between endothelial cell dysfunction and endothelial cell activation. (a) Cytokines and oxidized LDL stimulate endothelial cell permeability and NF-*κ*B-dependent inflammatory gene expression. ROS appear to play a central role mediating both responses. (b) In addition to its vasodilatory properties, NO promotes barrier stability, limits inflammation, inhibits platelet aggregation, and limits SMC proliferation. Loss of these protective properties in endothelial cell dysfunction therefore perpetuates endothelial cell activation.

The endothelial cell layer regulates multiple aspects of vascular physiology such as maintaining a semipermeable blood-tissue barrier, coordinating leukocyte trafficking, preventing thrombosis, and altering vascular tone [51]. During inflammatory responses, endothelial cells undergo a phenotypic conversion, termed endothelial cell activation, characterized by enhanced permeability, elevated leukocyte adhesion molecule expression, and reduced antithrombotic properties [52]. Mediators of endothelial cell activation are diverse and include proinflammatory cytokines (tumor necrosis factor *α* (TNF*α*), interleukin-1*β* (IL-1*β*)), bacterial (lipopolysaccharide) and endogenous toxins (oxLDL), vasoconstrictors (angiotensin II, endothelin-1), mechanical forces (shear stress, stretch), and extracellular matrix proteins (fibronectin, fibrinogen) [13, 52–54]. The early rapid phase of endothelial cell activation, termed type 1 activation, involves the remodeling of endothelial cell-cell junctions enhancing monolayer permeability (Figure 2(a)) and the export of Weibel-Palade bodies presenting P-Selectin and vWF at the endothelial cell surface [52]. Activation of proinflammatory transcription factors such as nuclear factor *κ*B (NF-*κ*B) drives the latter phase, termed type 2 activation, invoking the transcription and expression of proinflammatory genes such as E-selectin, ICAM-1, VCAM-1, chemokines (interleukin-8 (IL-8), monocyte chemotactic protein-1 (MCP-1)), and cytokines (IL-1*β*, TNF*α*) [52] (Figure 2(a)). The selectin family of cell adhesion molecules induces capture and tethering of leukocytes in the circulation resulting in leukocyte rolling across the endothelium. Rolling provides sufficient reduction in velocity for leukocyte firm adhesion to ICAM-1 and VCAM-1 on the endothelial surface and subsequent transendothelial migration [55]. Presentation of chemokines on the endothelial cell surface activates the leukocyte integrins VLA-4, LFA-1, and Mac-1 increasing their affinity for their ICAM-1/VCAM-1 ligands [55]. Transgenic mice lacking MCP-1, ICAM-1, or the fourth Ig domain of VCAM-1 (VCAM-1 expression is necessary for chorioallantoic fusion, rendering VCAM-1 knockouts lethal) severely limits atherosclerosis [56–60]. These data suggest that atherosclerotic plaque development requires endothelial cell activation involving enhanced expression of proinflammatory adhesion molecules and chemokines.

Endothelial cell dysfunction is defined by a decrease in the bioavailability of nitric oxide (NO), a critical regulator of vascular tone [61]. Multiple stimuli, including shear stress [62], acetylcholine [63], bradykinin [64], insulin [65, 66], and adiponectin [67], activate endothelial cell NO synthase (eNOS) to convert L-arginine into NO and citrulline. Endothelial cell dysfunction typically occurs when these protective stimuli are diminished, such as at sites of turbulent blood flow (decreased shear stress), in diabetes (decreased insulin signaling), and in obesity (decreased adiponectin). Decreases in eNOS activity and expression, increases in eNOS uncoupling, and direct NO scavenging all mediate endothelial cell dysfunction [61]. eNOS uncoupling appears to be particularly deleterious due to simultaneous decrease in NO production and increased production of the free radical superoxide. Multiple mechanisms have been proposed to mediate eNOS uncoupling, such as diminished bioavailability of L-arginine [68], accumulation of the endogenous eNOS inhibitor asymmetric dimethyl L-arginine (ADMA) [69], oxidation of the critical eNOS cofactor tetrahydrobiopterin [68, 70], and direct S-glutathionylation of critical cysteine residues in eNOS [71]. These modifications disrupt electron flow in eNOS causing incomplete catalysis of L-arginine and superoxide production [72, 73]. Superoxide can react with remaining intracellular NO further reducing cellular NO levels and resulting in the formation of peroxynitrite, a potent oxidant.

Endothelial cell dysfunction is regarded as an early step in atherosclerotic plaque formation primarily due to its effect on endothelial cell activation. Endothelial-derived NO reduces integrin activation on platelets [74, 75] and leukocytes [76] preventing thrombosis and leukocyte adhesion (Figure 2(b)). NO signaling in the endothelium promotes endothelial barrier integrity [77] and reduces both Weibel-Palade body exocytosis (type 1 activation) [78] and NF-*κ*B-dependent proinflammatory gene expression (type 2 activation) [79, 80] (Figure 2(b)). Turbulent flow, cytokines, and oxLDL reduce eNOS expression and shorten the half-life of eNOS mRNA perpetuating endothelial cell dysfunction and promoting inflammation (Figure 2(b)) [81]. Transgenic mice deficient in eNOS exhibit multiple vascular defects, such as hypertension, enhanced leukocyte rolling and firm adhesion, and vascular remodeling [82, 83]. Conversely, transgenic eNOS overexpression paradoxically *enhanced* atherosclerotic plaque formation due to eNOS uncoupling and superoxide production; supplementation with critical eNOS cofactor tetrahydrobiopterin reduced this uncoupling and diminished plaque formation in these mice [84]. Subsequent studies with tetrahydrobiopterin supplementation in ApoE mice demonstrated reduced plaque progression suggesting that eNOS uncoupling may be involved in atherosclerotic plaque progression in the absence of eNOS overexpression as well [85, 86]. Taken together, these data suggest a critical role for endothelial cell dysfunction in the propagation of both endothelial cell activation and atherosclerotic plaque formation.

## 3. Hyperglycemia in Endothelial Cell Dysfunction and Activation

The effect of hyperglycemia on endothelial cells closely mimics that of inflammatory initiators. Endothelial cells exposed to hyperglycemic cell culture media show reduced NO production [87, 88] with enhanced NF-*κ*B activation [89–91], inflammatory gene expression [92–95], and leukocyte recruitment [93, 94, 96]. Transient hyperglycemia causes epigenetic modifications in the promoter of the NF-*κ*B p65 subunit inducing a sustained increase in NF-*κ*B expression and NF-*κ*B-dependent VCAM-1 and MCP-1 expression for 6 days after restoration of normoglycemia [97], a phenomena termed epigenetic memory. Animal models and studies on human patients have similarly demonstrated an association between hyperglycemia and endothelial cell dysfunction and activation. However, it should be noted that most cell culture systems and animal models tend to utilize glucose levels (20–25 mM; 360 to 450 mg/dL) above that seen in diabetic humans (typically 7.8 to 10 mM; 140–180 mg/dL) to accelerate the occurrence of diabetic complications, and care must be taken when extrapolating results from these experimental systems to the human disease. In the subsequent sections, we will describe the roles of reactive oxygen species, advanced glycation end products (AGEs), metabolic pathway flux, and protein kinase C signaling in mediating the effects of hyperglycemia-induced endothelial cell dysfunction and activation.

### 3.1. Reactive Oxygen Species (ROS)

Multiple atherogenic stimuli promote endothelial cell dysfunction and activation through enhanced production of ROS. Several ROS play important roles in endothelial pathophysiology including the free radicals superoxide (O_2_^*⦁*−^) and the hydroxyl radical (OH^*⦁*^) as well as the non-free radical species hydrogen peroxide (H_2_O_2_), peroxynitrite (ONOO-/ONOOH), and hypochlorous acid (HClO) [98]. Turbulent flow [99, 100], atherogenic cytokines (TNF*α*, IL-1*β*) [101, 102], vasoconstrictors (angiotensin II) [103] and oxidized LDL [104] all induce ROS-dependent NF-*κ*B activation to drive endothelial cell expression of proinflammatory and prothrombotic genes [105, 106]. In addition to inflammatory gene expression, ROS reduce endothelial barrier function [107, 108] contributing to lipoprotein deposition and subsequent oxidative modification of LDL particles in the vessel wall [98, 109]. Furthermore, ROS stimulates endothelial cell dysfunction by scavenging NO directly or by oxidative modification of tetrahydrobiopterin resulting in eNOS uncoupling [110]. Thus, ROS reduce endothelial cell NO production (scavenging, eNOS uncoupling) while activating both direct (NF-*κ*B activation) and indirect mediators (LDL oxidation) of endothelial cell activation.

**Figure 3:**
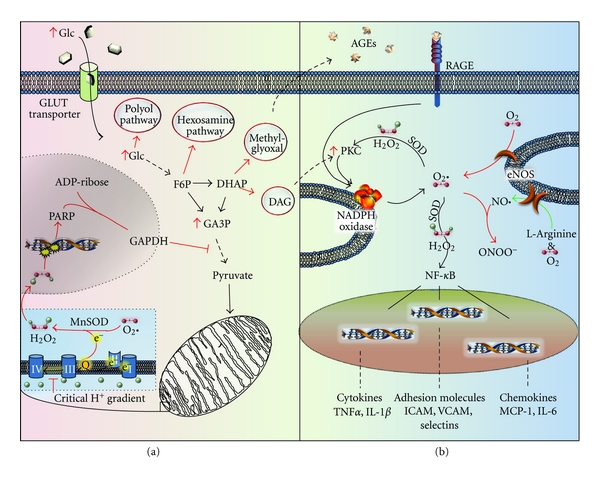
Hyperglycemia-induced endothelial dysfunction and activation. (a) Hyperglycemia induces metabolic dysfunction through mitochondrial production of superoxide, resulting in PARP activation and subsequent altered glycolytic flux to enhance diacylglycerol production (DAG), methylglyoxal production, and hexosamine and polyol pathway activity. (b) Hyperglycemia-induced oxidative stress is further enhanced by metabolic overproduction of DAG and decreases in NADH^+^/reduced glutathione (GSH), as well as stimulation of the RAGE receptor. Oxidative stress reduces protective mediators (NO bioavailability) and enhances inflammatory transcription factor (NF-*κ*B) activation resulting in inflammatory gene expression and leukocyte recruitment.

Hyperglycemia stimulates cellular ROS production by four major sources including direct glucose autooxidation [111], mitochondrial superoxide production [112], eNOS uncoupling [113], and AGE-dependent NADPH oxidase activation (Figure 3(a)) [72, 73]. Glucose autooxidation and mitochondrial superoxide are likely to be the initial contributors to ROS-mediated dysfunction elicited by hyperglycemia [72]. Trace amounts of free metals catalyze glucose auto-oxidation resulting in systemic oxidant stress [114]. Glucose oxidation during glycolysis produces ROS that are generally held in check by the cell's antioxidant defenses, including superoxide dismutase (SOD), thioredoxin, glutathione peroxidase (GP), and catalase [115]. However, these systems become overloaded during hyperglycemia. Whereas many cells downregulate glucose transporters (GLUTs) in response to hyperglycemia, endothelial cells retain expression of non-insulin-dependent GLUTs allowing intracellular glucose to rise concomitantly with extracellular glucose concentrations (Figure 3(a)) [116]. Enhanced glycolytic oxidation and the disruption of the mitochondrial electron transport chain promoting electron shuttling into molecular oxygen stimulates oxidant stress. eNOS uncoupling in response to ROS production further perpetuates oxidant stress under hyperglycemic conditions (Figure 3(b)) [117]. During chronic hyperglycemia, AGE production contributes to ROS production through receptor-mediated NADPH oxidase activation (Figure 3(b)). It should be noted however, that ROS production through distinct cellular sources (NADPH oxidase, mitochondria) can lead to ROS production at secondary sites [72, 118], and the relative contributions of each source are likely to fluctuate with disease state and control (hyperglycemic spikes versus AGEs).

Multiple studies have found that preventing ROS production or enhancing the cell's antioxidant systems limits the capacity of hyperglycemia to promote endothelial dysfunction and activation. Inhibiting mitochondrial superoxide production prevents hyperglycemia-induced ROS production, AGE accumulation, and PKC activation suggesting this pathway plays a central role in hyperglycemia-induced endothelial cell responses [119]. Addition of the antioxidant N-acetyl-L-cysteine prevented hyperglycemia-associated endothelial cell apoptosis [120], whereas the antioxidant coenzyme Q10 inhibits high-glucose-induced proinflammatory gene expression and monocyte binding [121]. Reducing hyperglycemia-induced ROS production using a mitochondrial complex II inhibitor (thenoyltrifluoroacetone (TTFA)) or a Mn SOD mimetic (Mn(III)tetrakis(4-benzoic acid) porphyrin chloride (MnTBAP)) abrogates inflammatory gene expression [122, 123] and enhances NO production [123]. Taken together, these studies suggest that limiting ROS production reduces hyperglycemia-induced endothelial cell dysfunction and activation.

Consistent with cell culture models, mouse models of type 1 diabetes show enhanced endothelial oxidative stress, inflammatory gene expression, and leukocyte recruitment [124–126]. Pharmacological and genetic modifications of antioxidant pathways support a role for oxidative stress in hyperglycemia-associated endothelial cell dysfunction and activation. Rings of rabbit aorta treated for 6 hours in hyperglycemic media show reduced ACh-mediated vasodilation compared to normoglycemic controls, and multiple antioxidants (superoxide dismutase, catalase, deferoxamine, or allopurinol) were sufficient to reverse hyperglycemia-associated impairment in vasodilation [127]. Similarly, multiple antioxidants restored vasodilation in aortic rings isolated from diabetic rats [128, 129]. Diabetic ApoE/glutathione peroxidase-1 double KO mice show enhanced atherosclerosis compared to diabetic mice deficient in ApoE alone associated with enhanced VCAM-1 expression and macrophage recruitment [130]. Conversely, the GP-1 mimetic ebselen reduces endothelial dysfunction, proinflammatory gene expression, and atherosclerotic plaque formation in diabetic mice [131, 132]. Antioxidant treatments also reduce endothelial permeability resulting from hyperglycemia [133, 134]. Taken together, these data provide strong evidence that antioxidants protect endothelial cells from hyperglycemia-induced endothelial cell dysfunction and activation.

Although many antioxidant therapies targeting multiple sources of ROS in experimental models have shown promise, several clinical trials utilizing antioxidants appear to confer no significant protection [73, 135]. In the Heart Outcomes Prevention Evaluation (HOPE) trial, 3654 diabetic patients receiving supplementation with vitamin E or placebo for 4.5 years failed to show any cardiovascular benefit for vitamin E [136]. Similarly, the Secondary Prevention with Antioxidants of Cardiovascular Disease in End Stage Renal Disease (SPACE) trial [137], Study to Evaluate Carotid Ultrasound Changes in Patients Treated with Ramipril and Vitamin E (SECURE) trial [138], and the Primary Prevention Project (PPP) trial [139] all failed to show beneficial effects of antioxidants in diabetes-associated cardiovascular disease. However, the lack of a clinical benefit in these trials does not disprove the role of ROS in CVD as there are several reasons why these studies may have failed including uncertain dose requirements for vitamin E, short time frame of clinical trials, and the use of high risk patients with advanced CVD [140].

### 3.2. AGE/RAGE

Another major mechanism of diabetes-associated endothelial cell dysfunction and activation involves hyperglycemia-associated formation of AGEs. Glycation involves the nonenzymatic addition of a carbohydrate moiety onto proteins; this should not be confused with glycosylation that takes place in the ER and Golgi resulting in protein maturation. The nonreversible formation of AGEs results from several reversible reactions, collectively termed the Maillard reaction. First, a reducing sugar, such as glucose or fructose, attaches to the *α*-amino group of either the amino terminus of proteins or lysine residues via nucleophilic attack: the product is known as a Schiff base [141]. This can undergo an Amadori rearrangement forming ketoamines. From here, these ketoamines, synonymously termed Amadori products, take one of two pathways: an oxidative or nonoxidative pathway forming irreversible AGE modifications [142, 143]. Hyperglycemia causes excessive glycation of proteins found in serum (e.g., albumin, hemoglobin, and LDL) and in the vessel wall (e.g., collagen, fibronectin). However, AGE formation by intracellular glycolysis-derived dicarbonyl precursors (glyoxal, methylglyoxal, and 3-deoxyglucosone) occurs at a rate several orders of magnitude higher than nonenzymatic glycation, suggesting that these intermediates may be primarily responsible for both intracellular and extracellular AGE production (Figure 3(b)) [144, 145]. Glyoxal arises from glucose autooxidation, whereas methylglyoxal formation occurs in response to glyceraldehyde-3-phosphate and dihydroxyacetone phosphate fragmentation [146–148]. While enhanced during hyperglycemia, AGE formation is a naturally occurring process with endogenous negative regulators (ex. glyoxalase I) that degrade the AGE-inducing dicarbonyl intermediates [146]. Interestingly, AGE accumulation during normal aging occurs concomitant with a decrease in glyoxalase I expression [149], and glyoxalase I expression is associated with enhanced lifespan of *Caenorhabditis elegans* [150].

AGEs carry a large arsenal of weaponry with which to exacerbate diseases, including both receptor-mediated and receptor-independent effects. AGEs bind to multiple cell surface receptors including the “receptor for AGE” (RAGE) [151], AGE receptor 1 (AGER1), AGER3, and CD36 [152]. A member of the immunoglobulin superfamily, RAGE regulates AGE-associated endothelial cell dysfunction and activation. While RAGE receptor signaling is not well understood, RAGE ligation stimulates endothelial ROS production [153], and the NADPH oxidase inhibitor diphenyliodonium (DPI) significantly blunts this induction (Figure 3(b)) [154]. AGE-dependent proinflammatory gene expression (VCAM-1, E-selectin) in endothelial cells similarly requires the RAGE receptor [155] and NADPH oxidase activation [154, 156]. In addition to ROS-mediated NO scavenging, AGEs decrease eNOS expression and L-citrulline production (readout of NO production) in endothelial cells [157], and AGEs inhibit histamine-induced NO production in endothelial cells associated with reductions in eNOS serine phosphorylation [158]. Consistent with this, Gao and collaborators show that the impaired vasodilation in blood vessels of diabetic mice is endothelium dependent and RAGE sensitive [159].

In contrast to the proinflammatory RAGE receptor, the alternative AGE receptors AGER1, AGER3, and CD36 stimulate AGE degradation [152], with AGER1 suppressing AGE-induced ROS production [160]. This disparity suggests that AGE-associated inflammation can be regulated at the level of receptor expression. Activation of NO-associated PKG signaling in endothelial cells by treatment with the PDE5 inhibitor Vardenafil reduces RAGE gene expression, suggesting that healthy levels of NO/PKG signaling suppress the AGE/RAGE insult [161]. In contrast, hyperglycemia-induced ROS production promote RAGE expression [162], suggesting a synergistic effect between hyperglycemia-associated glycolytic oxidant stress and AGE/RAGE-dependent oxidant stress.

Receptor-independent effects of AGEs include extracellular matrix modification [163], NO scavenging [164], and glycation of both signaling proteins [165] and LDL [166]. Since AGE formation is irreversible and turnover of ECM is slow, hyperglycemia stimulates considerable glycation of extracellular matrix proteins resulting in vessel stiffening through crosslinking type I collagen and elastin [167–169]. Vessel stiffening contributes to systemic hypertension and increases the strain on the vessel wall [170]. AGE-dependent NO scavenging perpetuates stiffening by enhancing smooth muscle proliferation and increased contractility [164, 171]. Endothelial cell interactions with subendothelial basement membrane proteins initiate signaling pathways that reduced endothelial cell activation [100, 172]. Glycation of the subendothelial matrix proteins laminin and collagen IV disrupts matrix self-assembly and prevents endothelial cell adhesion and spreading [169, 173, 174]. Therefore, disrupted interaction with the basement membrane may limit its protective properties while enhancing endothelial cell loss (superficial plaque erosion). In addition to matrix modifications, glycation of intracellular signaling proteins by methylglyoxal activates the endothelial cell stress response (JNK, p38) [175]. However, methylglyoxal inhibits NF-*κ*B activation by modification of Cys38 blocking DNA binding [165], and JNK signaling in the absence of NF-*κ*B can promote apoptosis [176]. Consistent with this, methylglyoxal stimulates endothelial cell apoptosis although the signaling mechanisms employed have not been addressed directly [177]. Lastly, glycation of LDL may be as deleterious as LDL oxidation in promoting endothelial cell activation [178]. Glycated LDL binds to the RAGE receptor stimulating endothelial cell dysfunction through calpain-dependent eNOS degradation and promoting inflammatory gene expression through NADPH oxidase-dependent ROS production [179–182].

Several lines of evidence suggest that AGE formation mediates hyperglycemia-associated cardiovascular disease. Pharmacological inhibitors of AGE formation (alagebrium chloride (ALT-711), pyridoxamine dihydrochloride) significantly reduce atherosclerotic plaque formation in diabetic ApoE null mice [183, 184]. Early clinical trials using ALT-711 demonstrated enhanced arterial compliance [185] and improved flow-mediated vasodilation [186] in aged hypertensive patients. Despite these early successes, Alteon (the producer of ALT-711) encountered financial hardship and halted drug development. Overexpression of methylglyoxal-degrading enzyme glyoxalase I reduces AGE production and oxidant stress in diabetic rats [187]. Furthermore, polymorphisms in glyoxalase I are associated with carotid atherosclerosis in type 2 diabetics [188, 189]. A soluble splice variant of RAGE lacking the cytosolic and transmembrane domain scavenges circulating AGEs counteracting their proinflammatory effects. Therapeutic intervention utilizing exogenous sRAGE treatment dose-dependently inhibits leukocyte infiltration, plaque formation, and progression of existing plaques in mouse models [190, 191]. One caveat to these studies is the interaction of RAGE with oxLDL, suggesting sRAGE may reduce atherosclerosis by scavenging oxLDL. However, ApoE/RAGE double knockout mice and ApoE mice expressing endothelial-specific dominant negative RAGE show reductions in both endothelial cell dysfunction and atherosclerotic plaque formation [192], suggesting that RAGE plays an important role in atherosclerotic plaque formation. RAGE antagonists (TTP488) and humanized sRAGE are making their way through early clinical trials for diabetic nephropathy, Alzheimer's disease, and acute lung injury; however no trials for cardiovascular disease are currently underway [193].

### 3.3. Metabolic Pathway Flux

In addition to uncoupling the electron transport chain, hyperglycemia pushes glucose flux through alternative metabolic pathways, including the polyol and hexosamine pathways (Figure 3(a)). The polyol pathway consists of aldose reductase and sorbitol dehydrogenase, which mediate the conversion of glucose to sorbitol via NADPH oxidation, and sorbitol to fructose via NAD^+^ reduction, respectively. In hyperglycemic conditions, it is suggested that up to 30% of metabolized glucose fluxes through the polyol pathway [194] versus the ~3% under normo-glycemic conditions [195]. Hyperglycemia stimulates aldose reductase expression and may affect activation through ROS-dependent S-thiolation, S-nitrosation, and glutathiolation of a critical cysteine residue (Cys298) [196, 197]. Early studies demonstrated that limiting aldose reductase activity in the lens inhibits cataract formation driven by sorbitol accumulation [196, 198–200]; thus aldose reductase was postulated to mediate peripheral hyperglycemic complications. This effect was proposed to be due to osmotic pressure caused by sorbitol accumulation. However, multiple antioxidants were later found to limit cataract formation under hyperglycemic conditions without affecting sorbitol accumulation suggesting a more complex role for aldose reductase in hyperglycemia-associated diabetic complications [196, 201–203].

Aldose reductase is expressed in multiple tissue beds and performs highly context-dependent functions [204]. Inhibiting aldose reductase limits NF-*κ*B activation and proinflammatory gene expression in response to hyperglycemia and proinflammatory cytokines, suggesting aldose reductase plays a proinflammatory function in endothelial cells [205, 206]. The proinflammatory effect of polyol pathway flux could be explained by the reduction of NADPH and NAD^+^ availability, resulting in reduced production of NO, diminished levels of reduced glutathione, and unbalanced redox stress [72]. However, aldose reductase also catalyzes the reduction of aldehydes produced by lipid peroxidation and glutathiolation [196, 207–209]. Since these aldehydes possess proinflammatory properties, aldose reductase may also function as an anti-inflammatory/detoxification mediator [209]. Mouse models of atherogenesis underscore the complexities of aldose reductase activity in the disease setting. Whereas aldose reductase deletion (global) enhances atherosclerotic plaque in the hypercholesterolemic ApoE-null mouse on high-fat diet as well as in streptozotocin-induced diabetes [210], general and endothelial targeted overexpression of human AR in models of hypercholesterolemia also showed enhanced plaque size in diabetic animals [211, 212]. This discrepancy may be due to the significantly reduced levels of aldose reductase in mice, which could limit polyol pathway flux and diminish its potential proinflammatory role [197].

In contrast to the extensive research on the polyol pathway in hyperglycemia, the hexosamine pathway has received considerably less attention, possibly owing to the lack of specific inhibitors [213, 214]. In the physiological setting, fructose-6-phosphate proceeds from the glycolytic pathway to produce glucosamine-6-phosphate via enzymatic activity of glutamine:fructose-6-phosphate amidotransferase (GFAT). Several intermediate reactions lead to synthesis of UDP-N-acetylglucosamine (GlcNAc), a nucleotide donor utilized in the ER and golgi for glycosylation of various glycoproteins, lipids, and proteoglycans. Additionally, UDP-GlcNAc is a substrate of O-GlcNAc transferase (OGT), leading to production of O-linked GlcNAcylated Ser and Thr residues in a wide variety of intracellular proteins [215, 216]. The regulation of many processes by O-GlcNAc modification has led to the comparison of O-GlcNAcylation to phosphorylation events and redox modification of proteins as a regulator of protein activity, with emphasis on the limited expression of enzymes involved in the regulation of O*-*GlcNAcylation versus the plethora of cellular kinases mediating phosphorylation events [217].

Protective roles of O-GlcNAcylation have been reported in cardiac ischemia/reperfusion [218, 219], and the use of glucosamine to bypass GFAT for UDP-GlcNAc production appears to serve anti-inflammatory roles [220, 221]. However, protective roles of O-GlcNAcylation may be context dependent. Indeed, hyperglycemia-induced O-GlcNAcylation of eNOS, dependent on the Ser1177 residue, occurs concomitant with decreased Ser1177 phosphorylation and reduced NO production [88, 222]. Endothelial O-GlcNAcylation is increased in carotid plaques of type II diabetic patients, and inhibition of the hexosamine pathway is sufficient to reverse O-GlcNAcylation-mediated eNOS inhibition in human coronary endothelial cells in hyperglycemic conditions [223]. O-GlcNAcylation stimulates activation of the p38, ERK, and JNK pathways in response to hyperglycemia and contributes to the reduced activation of the insulin receptor and IR substrate, phosphoinositol-3-kinase, and Akt [214, 223]. Additionally, hyperglycemia enhances O-GlcNAcylation of the Sp1 transcription factor regulating expression of the profibrotic growth factor TGF*β* in arterial endothelial cells [224] and smooth muscle cells [225]. However, no definitive data exists linking O-GlcNAcylation to enhanced atherosclerotic plaque formation in diabetic mice or human patients.

Despite the discovery and characterization of O-GlcNAcylation in 1986 [226] and OGT in 1990 [216], no mature therapeutics have been developed targeting hexosamine stress. Although the glutamine analogs 6-diazo-5-oxo-L-norleucine and azaserine (O-diazoacetyl-L-serine) are frequently used to study the hexosamine pathway by inhibiting GFAT activity, the role of GFAT in glycosylation, and the ubiquitous nature of O-GlcNAcylation make GFAT an unattractive therapeutic target. However, as the regulation and contexts of O-GlcNAcylation are further characterized, new insights may prove this pathway feasible for intervention of hyperglycemic distress. In contrast, several comprehensive reviews highlight the escalating, decades-long success story of aldose reductase inhibitors (ARIs) [227–229]. Although the majority of ARIs to date have been less effective in human patients than in experimental models or have exhibited unforeseen but mild/reversible side effects, the ARIs have consistently demonstrated improvements in peripheral measures including nerve conduction velocity and sensation, albuminurea, and peripheral blood flow. Consequently, pharmaceutical companies have developed increasingly more potent ARIs. Currently, ranirestat is undergoing phase III trials in Europe and the United States, and epalrestat has been used clinically in Japan for several years. While the bulk of these trials and other experimental data support a proinflammatory role for aldose reductase [209], no clinical trials to date have tested the effectiveness of ARIs in treating macrovascular complications of diabetes.

### 3.4. Protein Kinase C (PKC)

The PKC family of serine/threonine kinases regulates a plethora of cellular functions. Mammalian cells express multiple PKC isoforms divided into subfamilies termed classical PKCs (PKC*α*, PKC*β*I, PKC*β*II, PKC*γ*), novel PKCs (PKC*δ*, PKC*ε*, PKC*η*, PKC*θ*, PKC*μ*), and atypical PKCs (PKC*ζ*, PKC*λ*/*ι*) [230, 231]. Activation mechanisms differ between subclasses with both classical and novel PKC isoforms showing activation by diacylglycerol, phosphatidylserine, and phorbol esters (e.g., phorbol-12-myristate-13-acetate (PMA)), whereas only classical PKCs are sensitive to calcium due to the presence of a calcium binding domain in the N-terminus [230, 231]. Atypical PKCs are insensitive to calcium and diacylglycerol and are activated instead by the phosphoinositide 3-kinase (PI-3K)/phosphoinositide-dependent kinase (PDK1) pathway [230, 231]. Evidence for PKC activation in hyperglycemia has implicated two major pathways leading to PKC activity. First, high-glucose-induced mitochondrial superoxide production leads to poly(ADP-ribose) polymerase (PARP) activation, and subsequent modification and inactivation of GAPDH by ADP-ribose polymers (Figure 3(a)) [72, 232]. GAPDH inactivation results in accumulation of the upstream glycolysis intermediate glyceraldehyde-3 phosphate which can be converted to diacylglycerol promoting the activation of classical and novel PKCs. Second, ligation of the RAGE receptor by AGEs also stimulates PKC activation albeit through uncertain mechanisms (Figure 3(b)) [233].

Signaling through PKC regulates multiple cellular processes involved in endothelial cell dysfunction and activation. PKC phosphorylates eNOS on an inhibitory site (Thr495) blunting eNOS activity [234, 235] and reduces eNOS phosphorylation on the activating Ser1177 site [236, 237]. TNF*α* reduces eNOS protein stability through PKC*ζ* suggesting that PKC influences NO production through a variety of mechanisms [238]. Interestingly, a study of the porcine aorta demonstrated that PKC*ζ* showed enhanced activity in regions of disturbed flow prone to atherosclerotic plaque development [239]. Conversely, PKC*α* shows both positive and negative effects on eNOS activity and NO production depending upon environmental context [240, 241]. In addition to modulating eNOS activity, PKC induces endothelial monolayer permeability directly via phosphorylation of junctional proteins (e.g., occludin) [242, 243] and indirectly through enhanced expression of the permeability-inducing factors VEGF [244], endothelin-1 [245], and thrombin [246]. Multiple PKC isoforms can feed directly into the NF-*κ*B activation pathway regulating downstream proinflammatory gene expression [247, 248]. Classic PKC isoforms PKC*α* and PKC*β* mediate NF-*κ*B activation and expression of ICAM-1/VCAM-1 in response to 12/15-lipoxygenase and apolipoprotein CIII [249, 250]. In contrast, thrombin-induced NF-*κ*B activation requires the novel isoform PKC*δ* [251, 252]. The atypical isoform PKC*ζ* mediates TNF*α*-induced NF-*κ*B activation and ICAM expression [253]. Taken together, these data suggest that PKC isoforms play stimulus- and context-dependent roles in modulating endothelial dysfunction and activation.

A mounting body of evidence implicates PKC signaling in multiple modalities of hyperglycemia-induced endothelial cell dysfunction and activation. Hyperglycemia stimulates a PKC-dependent reduction in eNOS expression and NO production in retinal and aortic endothelial cells [254, 255]. In addition, the PKC activator PMA promotes NADPH oxidase-dependent ROS production, and inhibiting PKC limited high-glucose-induced ROS in endothelial cells suggesting that PKC may reduce NO levels by inducing ROS-dependent scavenging [256]. The general PKC inhibitor staurosporine and the classical PKC inhibitor Go6976 reduce hyperglycemia-associated endothelial monolayer permeability suggesting that hyperglycemia-induced PKC signaling promotes endothelial permeability [257, 258]. However, the effect of PKC signaling on endothelial permeability depends upon the isoform involved, as PKC*δ* signaling reduces endothelial permeability in coronary artery endothelial cells and is downregulated in the coronary artery of diabetic rats [259]. Both staurosporine and calphostin, an inhibitor of classic and novel PKC isoforms, block hyperglycemia-induced NF-*κ*B activation [92, 260], and a specific PKC*β* inhibitor (ruboxistaurin) prevents AGEs-induced ICAM-1 expression and leukocyte adhesion in HUVECs [261]. Similarly, the PKC*β* inhibitor LY379196 prevents hyperglycemia-induced NF-*κ*B activation [262], VCAM-1 expression [262], and apoptosis [263], suggesting PKC*β* may be an attractive target to limit diabetes-associated endothelial activation *in vivo*.

Like AGE/RAGE and ROS, PKC appears to regulate endothelial dysfunction/activation and atherosclerotic plaque formation in animal models. PKC*α* and PKC*β*II show enhanced expression and activation in the diabetic macrovasculature [264–267], and the general PKC inhibitor bisindolylmaleimide-I blunts hyperglycemia-induced leukocyte binding to mesenteric postcapillary venules *in vivo* [268]. PKC*β*/ApoE double knockout mice develop smaller atherosclerotic plaques than mice deficient in ApoE alone [269], and the PKC*β* inhibitor ruboxistaurin reduces retinal and renal complications in diabetic rats while enhancing ACh-induced aortic vasodilation [270, 271]. In line with these studies, transient hyperglycemia blunts endothelial-dependent vasodilation in otherwise healthy human subjects, and PKC*β* inhibition with ruboxistaurin restores normal endothelial function in these patients [272]. Furthermore, ruboxistaurin enhanced brachial artery flow-mediated dilation in type 2 diabetics [273] suggesting that this inhibitor may reduce macrovascular endothelial dysfunction.

## 4. Conclusions

Multiple pathways drive hyperglycemia-induced endothelial cell dysfunction and activation including enhanced glycolysis (ROS), the buildup of glycolytic intermediates (polyol pathway, hexosamine pathway, PKC activation, and AGE formation), and AGE-modification of proteins (ROS, PKC). Given the interdependent nature of these pathways, it is not surprising that inhibitors targeting one of these pathways profoundly affect hyperglycemia-induced alterations in endothelial cell function. But which pathways are more attractive targets for clinical intervention? Trials limiting polyol pathway flux, AGE production, and RAGE signaling have shown a significant improvement in endothelial cell function; however these are not being actively pursued to treat CVD. The PKC*β* inhibitor ruboxistaurin shows great promise to reduce microvascular complications of diabetes and early results suggest an improvement in endothelial cell function in CVD patients. However, no trials to date have examined the effects of ruboxistaurin on diabetes-associated atherosclerotic burden directly. Prevention of early ROS production using mitochondrial complex II inhibitors blocks downstream AGE production and protects endothelial cells from transitioning to the dysfunctional or activated phenotypes [119]. However, clinical trials to target these early ROS-dependent pathways have shown less promising results than those targeting AGE formation or PKC signaling. Considering the clinical trial data concerning hyperglycemia itself, the major issue with these studies may simply involve timing of intervention. Clinical trials for antioxidants typically utilize patients already at a high risk for CVD, and interventions for hyperglycemia in these patients showed a similar lack of beneficial effect. The use of antioxidants early following the diagnosis of diabetes, especially coupled with intensive glycemic control, may provide additional benefit later in life. Consistent with this idea, intensive treatment to lower hyperglycemia in the DCCT and UKPDS trials demonstrate the most striking benefit in long-term follow-up studies 10 years after cessation of differential treatment; no long-term follow-up studies have been performed for antioxidant therapies. Therefore, more research is needed in this area if we are to translate the volumes of cell and molecular biology, animal research, and clinical trials into effective therapeutic strategies.
